# What do Cochrane systematic reviews say about probiotics as preventive interventions?

**DOI:** 10.1590/1516-3180.2017.0310241017

**Published:** 2017-09-28

**Authors:** Vinícius Lopes Braga, Luana Pompeu dos Santos Rocha, Daniel Damasceno Bernardo, Carolina de Oliveira Cruz, Rachel Riera

**Affiliations:** I Undergraduate Medical Student, Escola Paulista de Medicina (EPM), Universidade Federal de São Paulo (Unifesp), São Paulo (SP), Brazil; II Undergraduate Medical Students, Escola Paulista de Medicina (EPM), Universidade Federal de São Paulo (Unifesp), São Paulo (SP), Brazil.; III MD, MSc. Psychologist, Postgraduate Student, Evidence-Based Health Program, Universidade Federal de São Paulo (Unifesp), and Assistant Researcher, Cochrane Brazil, São Paulo (SP), Brazil; IV MD, PhD. Rheumatologist, Adjunct Professor, Discipline of Evidence-Based Medicine, Escola Paulista de Medicina (EPM), Universidade Federal de São Paulo (Unifesp), and Assistant Coordinator, Cochrane Brazil, São Paulo (SP), Brazil

**Keywords:** Review [publication type], Probiotics, Preventive medicine, Evidence-based medicine, Evidence-based practice

## Abstract

**BACKGROUND:**

Probiotics have been used for a range of clinical situations and their use is strongly encouraged by the media worldwide. This study identified and summarized all Cochrane systematic reviews about the preventive effects of probiotics in clinical practice.

**DESIGN AND SETTING:**

Review of systematic reviews, conducted in the Discipline of Evidence-Based Medicine, Escola Paulista de Medicina (EPM), Universidade Federal de São Paulo (Unifesp).

**METHODS:**

We included all Cochrane reviews on any probiotics when they were used as preventive interventions and compared with no intervention, placebo or any other pharmacological or non-pharmacological intervention.

**RESULTS:**

17 Cochrane systematic reviews fulfilled our inclusion criteria and were summarized in this report. None of the reviews included in the present study provided high-quality evidence for any outcome. The benefits from use of probiotics included decreased incidence of antibiotic-associated diarrhea and *Clostridium difficile*-associated diarrhea; decreased incidence of upper respiratory tract infections and duration of episodes; decreased need for antibiotics and absences from school due to colds; and decreased incidence of ventilator-associated pneumonia. Probiotics seem to decrease the incidence of gestational diabetes mellitus, birthweight, risk of vaginal infection and incidence of eczema.

**CONCLUSION:**

Despite the marketing and the benefits associated with probiotics, there is little scientific evidence supporting the use of probiotics. None of the reviews provided any high-quality evidence for prevention of illnesses through use of probiotics. More trials are needed to gain better knowledge of probiotics and to confirm when their use is beneficial and cost-effective.

## INTRODUCTION

More than 400 species of microorganisms dwell in the human gastrointestinal tract.^1^^,^^2^ Balance between them is vital for the host’s health. Present-day high usage of antibiotics, together with environmental and physiological factors, can alter this ecosystem. This imbalance can cause illnesses such as diarrhea, which was responsible for 1.31 million deaths in 2015, including 499,000 among children under five.^3^^,^^4^ Some research has shown that use of probiotics can confer some health benefits, such as treatment for diarrheal disease, prevention of systemic infections and other effects.^2^^,^^5^

The 2001 definition from the World Health Organization (WHO) states that probiotics are “live microorganisms which, when administered in adequate amounts, confer a health benefit on the host”.^6^ They are currently presented by media sources as an attractive health promotion method that prevents or cures a range of clinical situations.^7^ Indeed, many trials assessing the effects of probiotics (including using different species of microorganisms) as preventive or therapeutic options for a range of diseases have been conducted and published.^2^^,^^5^ Consequently, a considerable amount of published data is currently available through MEDLINE. Corroborating this, a search in this database carried out on July 26, 2017, using the MeSH (medical subheading) term probiotics, retrieved 12,370 records, which corresponded to an increase of 278% in the number of records over the last ten years (from December 2007 to July 2017).

The questions that therefore arise are: Should probiotics be indicated for preventive purposes? And if so, for which patients? Which types of probiotics should be used, and at what dose and for how long?

In this review, we identified and summarized all Cochrane systematic reviews about the preventive effects of probiotics in clinical practice.

## OBJECTIVE

To summarize the evidence from Cochrane systematic reviews focusing on probiotics for prevention of any disease or condition.

## METHODS

### Design

Review of Cochrane systematic reviews.

### Setting

Discipline of Evidence-Based Medicine of Escola Paulista de Medicina (EPM), Universidade Federal de São Paulo (UNIFESP). 

### Criteria for including reviews

- Types of studies We only included the latest version of completed Cochrane systematic reviews (SR). We excluded any published protocols or any SR marked as “withdrawn” in the Cochrane Database of Systematic Reviews (CDSR).
- Types of participants We included healthy participants or those diagnosed with any clinical condition or disease.
- Types of intervention This review included any probiotics that were used as preventive interventions and compared with no intervention, placebo or any other pharmacological or non-pharmacological intervention.
- Type of outcomes We considered any clinical, social and laboratory outcomes, as evaluated in the systematic reviews that were included.

### Search for reviews

We carried out a sensitive systematic search in the Cochrane Database of Systematic Reviews (via Wiley) on July 1, 2017. The search strategy is presented in Table 1.

**Table 1:** Search strategy (July 1, 2017)

#1 “Probiotics” OR “Probiotic” OR “lactobacillus” OR “lactobacilus” OR “lactobacilli” OR “lactobacili” OR “betabacterium” OR “lactobacileae” OR “lactobacilleae” OR “lactobacteria” OR “Lactobacillus acidophilus” OR “Lactobacilus acidophilus” OR “Lactobacillus casei” OR “Lactobacilus casei” OR “Lactobacillus delbrueckii” OR “Lactobacilus delbrueckii” OR “Lactobacillus fermentum” OR “Lactobacilus fermentum” OR “Lactobacillus helveticus” OR “Lactobacilus helveticus” OR “Lactobacillus leichmannii” OR “Lactobacilus leichmannii” OR “Lactobacillus plantarum” OR “Lactobacilus plantarum” OR “Lactobacillus reuteri” OR “Lactobacilus reuteri” OR “Lactobacillus rhamnosus” OR “Lactobacilus rhamnosus” OR “bifidobacterium” OR “lactococcus” OR “streptococcus thermophilus” OR “saccharomyces” OR “bifidobacterium” OR “bacillus subtilis” OR “bacillus licheniformis” OR “bugarian bacillus” OR “enterococcus faecalis” (Probiotics) OR (Probiotic) OR (lactobacillus) OR (lactobacilus) OR (lactobacilli) OR (lactobacili) OR (betabacterium) OR (lactobacileae) OR (lactobacilleae) OR (lactobacteria) OR (Lactobacillus acidophilus) OR (Lactobacilus acidophilus) OR (Lactobacillus casei) OR (Lactobacilus casei) OR (Lactobacillus delbrueckii) OR (Lactobacilus delbrueckii) OR (Lactobacillus fermentum) OR (Lactobacilus fermentum) OR (Lactobacillus helveticus) OR (Lactobacilus helveticus) OR (Lactobacillus leichmannii) OR (Lactobacilus leichmannii) OR (Lactobacillus plantarum) OR (Lactobacilus plantarum) OR (Lactobacillus reuteri) OR (Lactobacilus reuteri) OR (Lactobacillus rhamnosus) OR (Lactobacilus rhamnosus) OR (bifidobacterium) OR (lactococcus) OR (streptococcus thermophilus) OR (saccharomyces) OR (bifidobacterium) OR (bacillus subtilis) OR (bacillus licheniformis) OR (bugarian bacillus) OR (enterococcus faecalis) *in Title, Abstract, Keywords* #2 #1 *in Cochrane Reviews (complete*)

### Selection of systematic reviews

Two of the three researchers (VLB, LPDSR and DDB) independently and randomly selected and evaluated all references that were retrieved through the systematic search, to confirm their eligibility in accordance with the inclusion criteria. Any disagreements were resolved by consulting a more experienced author (RR).

### Presentation of the results

We presented all the reviews included in this synthesis in a narrative manner (qualitative synthesis). The key points considered were their relevance, methods, results, quality of the body of the evidence for each outcome, and applicability.

## RESULTS

### Search results

The initial search resulted in 39 reviews and 13 protocols. First, we excluded all protocols. After full-text assessment, we excluded 23 reviews since they either considered use of probiotics to be therapeutic interventions rather than preventive interventions or did not analyze probiotics alone. Thus, 16 Cochrane systematic reviews fulfilled our inclusion criteria and were summarized in this report.

### Results from systematic reviews

Among the 16 systematic reviews included, a range of probiotic strains was used. Four systematic reviews tested their use only among adults,^8^^,^^9^^,^^10^^,^^11^ three only among children^12^^,^^13^^,^^14^ and five among both adults and children,^15^^,^^16^^,^^17^^,^^18^^,^^19^ while another four studies did not specify the age range of the population evaluated.^20^^,^^21^^,^^22^^,^^23^ Two systematic reviews addressed prevention of respiratory diseases,^11^^,^^15^ nine addressed prevention of gastroenterological diseases,^9^^,^^10^^,^^13^^,^^14^^,^^16^^,^^17^^,^^18^^,^^20^^,^^21^ three addressed gynecological and obstetric diseases,^8^^,^^22^^,^^23^ one addressed urological diseases^19^ and one addressed immunological/allergic diseases.^12^ A summary of the reviews included is presented below. The main findings for each comparison and the quality of the evidence (based on the GRADE approach) are presented in Table 2. ^8^^,^^9^^,^^10^^,^^11^^,^^12^^,^^13^^,^^14^^,^^15^^,^^16^^,^^17^^,^^18^^,^^19^^,^^20^^,^^21^^,^^22^^,^^23^

**Table 2:** Characteristics, main findings and quality of evidence from systematic reviews focusing on patient-directed interventions with probiotics

Population and aim	Comparison	Benefits and harm of probiotics	Quality of evidence (GRADE approach)
Pediatric patients receiving antibiotics^13^	Probiotics versus placebo	Benefits: decreased incidence of antibiotic-associated diarrhea	Moderate
		Risk of some adverse effects in immunocompromised, severely debilitated and other patients	
Adults and children receiving antibiotics^17^	Probiotics versus placebo or no treatment	Benefit: decreased incidence of adverse events relating to *Clostridium difficile*-associated diarrhea	Moderate
		No benefit regarding incidence of *Clostridium difficile* infection	
Adults, children and the elderly^15^	Probiotics versus placebo	Benefit: decreased incidence of upper respiratory tract infections, duration of episodes, need for antibiotics and missing school due to colds	Very low to low
		No difference in adverse events	
Patients undergoing liver transplantation^16^	Prebiotics alone or plus probiotics versus selective bowel decontamination; Prebiotics plus probiotics versus prebiotics	Benefits: prebiotics plus probiotics decreased the proportion of participants with infections and the number of infectious episodes	Very low
		No benefit regarding mortality, need for retransplantation, graft rejection, intensive care unit stay or hospital stay	
Patients after liver resection^9^	Probiotics alone or plus prebiotics versus placebo or prebiotics plus probiotics postoperatively	No benefit regarding mortality	Low
Patients with quiescent ulcerative colitis^20^	Probiotics versus placebo or mesalazine	No benefit regarding prevention of relapses and adverse events	Low
Patients undergoing ileal pouch-anal anastomosis for chronic ulcerative colitis^10^	Probiotics versus placebo or other treatment	No benefit regarding prevention of pouchitis.	Very low
Patients susceptible to urinary tract infection and healthy people^19^	Probiotic versus placebo; probiotics versus antibiotics; probiotic versus no treatment	No benefit regarding symptomatic bacterial urinary tract infection	Low
Patients receiving mechanical ventilation^11^	Probiotics versus placebo	Benefit: decreased incidence of ventilator-associated pneumonia	Very low to low
		No effects regarding intensive care unit mortality, in-hospital mortality, incidence of diarrhea, length of intensive care unit stay, duration of mechanical ventilation or antibiotic use	
Women with HIV-infection for prevention of vulvovaginal candidiasis^23^	Probiotics versus placebo or clotrimazole	No benefit regarding prevention of vulvovaginal candidiasis infection	Low
Infants with family history of allergy or food hypersensitivity and healthy infants^12^	Probiotics alone or plus prebiotics versus placebo	Benefit: decreased incidence of infant eczema	Not assessed
		No benefit regarding food hypersensitivity, asthma, atopic eczema, allergic rhinitis, food allergy or urticaria	
Pregnant women without metabolic or chronic diseases^8^	Probiotics versus placebo or diet	Benefit: probiotics decreased both the rate of gestational diabetes mellitus and the birthweight	Not assessed
		No benefit regarding death (abortion, intrauterine fetal death, stillbirth or neonatal death), risk of premature birth or cesarean delivery rate	
Patients with Crohn’s disease^18^	Probiotics versus placebo or other treatment	No benefit regarding reduction of the risk of relapse after surgically-induced remission, compared with use of aminosalicylates or azathioprine.	Not assessed
		Risk of some adverse effects from *Lactobacillus GG*	
Infants born at gestational age of less than 37 weeks or weighing less than 2500 g at birth, or both^14^	Probiotics versus placebo or no treatment	Benefit: decreased incidence of severe necrotizing enterocolitis and mortality among preterm infants	Not assessed
		No effect regarding nosocomial sepsis	
Patients undergoing surgery related to Crohn’s disease^21^	Probiotics versus placebo	No benefit regarding clinical recurrence, severe endoscopic recurrence or any endoscopic recurrence	Not assessed
Pregnant women^22^	Probiotics versus placebo or acetic acid	Benefit: decreased risk of vaginal infection	Not assessed
		No effect regarding prevention of preterm birth	

### Moderate quality of evidence

#### Antibiotic-associated diarrhea among children

The review^13^ assessed the efficacy and safety of probiotics for prevention of antibiotic-associated diarrhea (AAD) among children and included 23 RCTs (938 children, aged 0 to 18 years) that compared different types of probiotics versus active treatment, placebo or no treatment. The incidence of antibiotic-associated diarrhea was lower in the probiotic group (relative risk, RR 0.46; 95% CI 0.35 to 0.61; number needed to treat [NNT] 10; 22 RCTs; 3,898 participants; I^2^ = 55%; moderate quality of evidence). No adverse events attributable to the treatment were found (2455 participants; 16 RCTs) and there was no difference between probiotics and controls regarding the risk of adverse events for the overall population (risk difference [RD] 0.00; 95% CI -0.01 to 0.01; 16 RCTs; 2455 participants). The authors concluded that there was moderate quality of evidence that probiotics seemed to prevent AAD. However, in some groups of patients such as immunocompromised individuals and others, serious events have been observed, and therefore probiotics should be avoided in these groups. For further details, and to check the probiotics used in each study, refer to the original abstract, available at: http://onlinelibrary.wiley.com/doi/10.1002/14651858.CD004827.pub4/full.

#### Clostridium difficile-associated diarrhea

The review^17^ evaluated the efficacy and safety of probiotics for prevention of *Clostridium difficile*-associated diarrhea (CDAD) and included 31 RCTs (4,492 adults and children), with comparisons between probiotics and placebo or no treatment. Probiotics presented benefits for reducing the incidence of CDAD (risk ratio [RR] 0.36; 95% CI 0.26 to 0.51; 23 RCTs; 4,213 participants, moderate quality of evidence) and of adverse events (RR 0.80; 95% CI 0.68 to 0.95; 26 RCTs; 3,964 participants; moderate quality of evidence). There was no statistical difference between the groups regarding the incidence of *C. difficile* infection (RR 0.89; 95% CI 0.64 to 1.24; 13 RCTs; 961 participants; moderate quality of evidence). The authors concluded that, based on the moderate quality of evidence, use of probiotics seemed to be associated with reduction of CDAD and its adverse events. For further details, and to check the form of probiotics used in each study, refer to the original abstract, available at: http://onlinelibrary.wiley.com/doi/10.1002/14651858.CD006095.pub3/full.

### Very low to low quality of evidence

#### Acute upper respiratory tract infections

The review^15^ evaluated the effectiveness and safety of probiotics for prevention of acute upper respiratory tract infections (URTIs) and included 13 RCTs (3,780 participants) that compared probiotics with placebo. Probiotics presented benefits through reducing the following outcomes:

- the number of people who had one or more URTIs (odds ratio, OR 0.53; 95% confidence interval, CI 0.37 to 0.76; 7 RCTs; 1,927 participants; low quality of evidence);
- the number of people who had three or more URTIs (OR 0.53; 95% CI 0.36 to 0.80; three RCTs; 650 participants; low quality of evidence);
- the duration of the event (mean difference [MD] -1.89; 95% CI -2.03 to -1.75; 3 RCTs; 831 participants; low quality of evidence);
- the need for antibiotics (OR 0.65; 95% CI 0.45 to 0.94; 4 RCTs; 1,184 participants; moderate quality of evidence); and
- missing school due to colds (OR 0.10; 95% CI 0.02 to 0.47; 1 RCT; 80 children; very low quality of evidence).

No difference between the groups was found regarding adverse events (OR 0.88; 95% CI 0.65 to 1.19; 4 RCTs; 1,234 participants; low quality of evidence). The authors concluded that, based on very low to low quality of evidence due to the heterogeneity between studies, use of probiotics may be associated with reductions in the numbers of URTIs, duration of the event, need for antibiotics and missing school due to URTIs. For further details, and to check the form of probiotics used in each study, refer to the original abstract, available at: http://onlinelibrary.wiley.com/doi/10.1002/14651858.CD006895.pub3/full.

#### Bacterial sepsis and wound complications after liver transplantation

The review^16^ assessed the effects of different interventions for prevention of bacterial sepsis and wound complications in patients undergoing liver transplantation. Seven RCTs were included in this review, but only two (161 participants) assessed preventive effects of probiotics. There was no difference between probiotics plus prebiotics and selective bowel decontamination regarding the risk of needing retransplantation (OR 2.91; 95% CI 0.12 to 68.81; 1 RCT; 63 participants) or the risk of graft rejection requiring medical treatment (OR 1.94; 95% CI 0.38 to 9.83; 1 RCT; 63 participants). In comparing probiotics plus prebiotics with prebiotics, there was no difference regarding the risk of retransplantation (OR 0.33; 95% CI 0.01 to 7.90; 2 RCTs; 129 participants) or the risk of graft rejection requiring medical treatment (OR 0.69; 95% CI 0.12 to 3.84; 1 RCT; 63 participants). The authors’ conclusion was that there was no evidence to support use of probiotics for reducing wound complications and bacterial sepsis in patients with previous liver transplantation. For further details, and to check the probiotics used in each study, refer to the original abstract, available at: http://onlinelibrary.wiley.com/doi/10.1002/14651858.CD006660.pub3/full.

#### Infections after liver resection

The review^9^ assessed the benefits and harm of different interventions for prevention of infectious complications and improving the outcomes after liver resection. Seven RCTs were included, but only two RCTs (125 participants) evaluated probiotics as preventive interventions. One of these compared use of prebiotics and probiotics versus placebo and found that there was no difference in mortality (RR 0.36; 95% CI 0.10 to 1.35; 44 participants). The other RCT, with 81 participants, compared use of preoperative and postoperative prebiotics and probiotics versus use of postoperative prebiotics and probiotics and found that there was no significant difference in mortality (RR 0.39; 95% CI 0.15 to 1.00). Both of these studies presented low quality of evidence.

The authors’ conclusion was that there was no evidence to support or refute the use of any type of treatment to decrease the frequency of infectious complications after liver resection. For further information, refer to: http://onlinelibrary.wiley.com/doi/10.1002/14651858.CD006933.pub2/full.

#### Maintenance of remission in ulcerative colitis

The review^20^ assessed the efficacy and safety of probiotics for prevention of relapses in cases of ulcerative colitis. Four RCTs (587 participants) were included and these showed the following: probiotics versus mesalazine: no difference between the groups regarding the risk of relapse (OR 1.33; 95% CI 0.94 to 1.90; 3 RCTs; 555 participants; low quality of evidence) or the incidence of adverse events (OR 1.21; 95% CI 0.80 to 1.84; 2 RCTs; 430 participants; moderate quality of evidence); probiotics versus placebo: no difference between the groups regarding the risk of relapse (OR 0.27; 95% CI 0.03 to 2.68; 1 RCT; 32 participants; moderate quality of evidence). The authors concluded that there was insufficient evidence to support use of probiotics for preventing relapses in cases of ulcerative colitis. For further details, and to check the form of probiotics used in each study, refer to the original abstract, available at: http://onlinelibrary.wiley.com/doi/10.1002/14651858.CD007443.pub2/full.

#### Pouchitis after ileal pouch-anal anastomosis for ulcerative colitis 

The review^10^ assessed the effectiveness of different interventions for prevention of pouchitis after ileal pouch-anal anastomosis in cases of chronic ulcerative colitis. Thirteen RCTs were included in this review, but only two studies were about the use of probiotics. One RCT assessed prevention of pouchitis in patients with ileal pouch-anal anastomosis and showed that there were no benefits from using *Bifidobacterium longum*, in comparison with placebo (RR 1.43; 95% CI 0.66 to 3.11; 1 RCT; 12 participants; very low quality of evidence).

Another outcome assessed related to treatment of acute pouchitis. One RCT with 20 participants compared the use of *Lactobacillus GG* with placebo and there was no difference in clinical improvement (RR 3.95%; CI 0.14 to 65.9), with very low quality of evidence.

The authors’ conclusions, based on the very low quality of evidence, was that probiotics did not seem to prevent pouchitis after ileal pouch-anal anastomosis in cases of chronic ulcerative colitis or to have any effect in treatments for patients with acute pouchitis. For more information about the other types of treatment in this study, refer to this link: http://onlinelibrary.wiley.com/doi/10.1002/14651858.CD001176.pub3/full.

#### Urinary tract infections

The review^19^ evaluated the effects of probiotics for prevention of urinary tract infections in susceptible or healthy adults and children. Nine RCTs (735 participants) were included and these showed that there was no difference in the risk of symptomatic bacterial urinary tract infection, in comparisons of use of probiotics versus placebo (OR 0.82; 95% CI 0.60 to 1.12; 6 RCTs; 352 participants) or use of probiotics versus antibiotics (OR 1.12; 95% CI 0.95 to 1.33; 1 RCT; 158 women).

The authors concluded that, based on the few studies available, which were of small size (low quality of evidence), there was no benefit from using probiotics, in comparison with placebo or no treatment. For further details, and to check the probiotics used in each study, refer to the original abstract, available at: http://onlinelibrary.wiley.com/doi/10.1002/14651858.CD008772.pub2/full.

#### Ventilator-associated pneumonia

The review^11^ assessed the effects of probiotics for prevention of ventilator-associated pneumonia (VAP). Eight randomized clinical trials (RCTs) (1,083 adults) that compared use of probiotics with placebo, usual care and multiple treatment arms were included. Probiotics were shown to present some benefit regarding reduction of the incidence of VAP (odds ratio, OR 0.70; 95% CI 0.52 to 0.95; 8 RCTs; 1,018 participants; low quality evidence). No difference was found between the probiotics and control groups in relation to the following outcomes,:

- intensive care unit (ICU) mortality (OR 0.84; 95% CI 0.58 to 1.22; 5 RCTs; 703 participants; very low quality of evidence);
- in-hospital mortality (OR 0.78; 95% CI 0.54 to 1.14; 4 RCTs; 524 participants; very low quality of evidence);
- incidence of diarrhea (OR 0.72; 95% CI 0.47 to 1.09; 4 RCTs; 618 participants; low quality of evidence);
- length of ICU stay (mean difference, MD -1.60; 95% CI -6.53 to 3.33; 4 RCTs; 396 participants; very low quality of evidence);
- duration of mechanical ventilation (MD -6.15; 95% CI -18.77 to 6.47; 2 RCTs; 203 participants; very low quality of evidence); and
- antibiotic use (OR 1.23; 95% CI 0.51 to 2.96; 1 RCT; 259 participants; low quality of evidence).

The authors concluded that, based on the low quality of evidence, use of probiotics may be associated with reduction in VAP. For further details, and to check the probiotics used in each study, refer to the original abstract, available at: http://onlinelibrary.wiley.com/doi/10.1002/14651858.CD011513.pub2/abstract.

#### Vulvovaginal candidiasis in HIV-infected women

The review^23^ aimed to assess the effects of many antifungals that are administered vaginally or orally, including probiotics, for treatment and prevention of vulvovaginal candidiasis (VVC) in HIV-infected women. Two trials (431 participants) on use of probiotics as preventive interventions were included. No difference regarding this outcome was found in comparisons of probiotics versus clotrimazole (RR 1.11; 95% CI 0.45 to 2.76; low quality of evidence) or versus placebo (RR 0.54; 95% CI 0.26 to 1.13; low quality of evidence). The authors’ conclusion was that no implications for practice could be determined. For other results from this review, refer to this link: http://onlinelibrary.wiley.com/doi/10.1002/14651858.CD008739.pub2/full.

### Quality of evidence not assessed

#### Allergic disease and food hypersensitivity among children

The review^12^ assessed the effect of probiotics for prevention of allergic disease relating to food hypersensitivity among infants. It included 12 RCTs that compared use of probiotics with placebo or use of probiotics plus prebiotics with placebo. The overall results from the pooled data showed that the incidence of infant eczema was reduced in the probiotics group (RR 0.82; 95% CI 0.70 to 0.95; 5 RCTs; 1,477 participants; I^2^ = 63.6%). However, these studies were heterogeneous and there was no statistical difference when the analysis was limited to atopic eczema (RR 0.80; 95% CI 0.62 to 1.02). There was also no significant difference regarding the other outcomes evaluated. The authors concluded that there was insufficient evidence to support use of probiotics for preventing allergies or food hypersensitivity among infants, given that the findings were inconsistent and there were many follow-up losses. For further details, and to check the form of probiotics used in each study, refer to the original abstract, available at: http://onlinelibrary.wiley.com/doi/10.1002/14651858.CD006475.pub2/full.

#### Gestational diabetes in women without metabolic or chronic diseases

The review^8^ evaluated the effects of probiotics for prevention of gestational diabetes mellitus (GDM). One RCT (256 pregnant women) was included and it found that there were benefits from use of probiotics (compared with placebo or diet) for reducing the rate of GDM (RR 0.38; 95% CI 0.20 to 0.70; 225 women) and for reducing the birthweight (MD -127.71 g; 95% CI -251.37 to -4.06; 256 women). No difference between the groups was found for the following outcomes:

- death (OR 2.00; 95% CI 0.35 to 11.35; 256 women);
- risk of premature birth (RR 3.27; 95% CI 0.44 to 24.43; 238 women);
- cesarean delivery (RR 1.23; 95% CI 0.65 to 2.32; 218 women).

All the infants included in this study were within the normal range for birthweight. The authors concluded that use of probiotics seemed to be associated with reduction in GDM. However, they considered that further studies would be required to confirm these results. For more details, and to check the form of probiotics used in each study, refer to the original abstract, available at: http://onlinelibrary.wiley.com/doi/10.1002/14651858.CD009951.pub2/full.

#### Maintenance of remission of Crohn’s disease

The review^18^ assessed the effectiveness of probiotics for maintenance of remission in cases of Crohn’s disease. Seven RCTs, comparing use of probiotics versus placebo, were included. In evaluating the risk of relapse among adults, there were no differences between the following:

- *E. coli* Nissle and placebo (RR 0.43; 95% CI 0.15 to 1.20; 1 RCT; 20 participants);
- *Lactobacillus GG* and placebo after surgically-induced remission (RR 1.58; 95% CI 0.30 to 8.4; 1 RCT; 37 participants) or medically-induced remission (RR 0.83; 95% CI 0.25 to 2.80; 1 RCT; 9 participants);
- *Lactobacillus GG* and maintenance therapy with aminosalicylates or azathioprine (RR 0.67; 95% CI 0.13 to 3.30; 1 RCT; 24 participants).

Regarding the risk of relapse among children, there was no difference between *Lactobacillus GG* and placebo (RR 1.85; 95% CI 0.77 to 4.40; 1 RCT; 75 participants).

Regarding the risk of adverse events, *Lactobacillus GG* was associated with a risk of adverse events in comparison with maintenance therapy using aminosalicylates or azathioprine (no numerical data provided). A small RCT found that, at the end of the study, there was no difference in use of *Saccharomyces boulardii* plus conventional maintenance therapy versus placebo plus conventional maintenance therapy for relapses, according to the clinical disease activity index (CDAI) (RR 0.17; 95% CI 0.02 to 1.23). 

The authors concluded that there was no evidence that probiotics were beneficial for maintenance of remission in cases of Crohn’s disease. For further details, and to check the probiotics used in each study, refer to the original abstract, available at: http://onlinelibrary.wiley.com/doi/10.1002/14651858.CD004826.pub2/full.

#### Necrotizing enterocolitis in premature newborns

The review^14^ assessed the efficacy and safety of probiotics for prevention of severe necrotizing enterocolitis (NEC) or sepsis in premature infants. Twenty-four RCTs, comparing use of probiotics with placebo or no treatment, were included. Probiotics showed some benefit regarding reduction of the incidence of severe NEC (RR 0.43; 95% CI 0.33 to 0.56; 20 RCTs; 5,529 infants) and all-cause mortality (RR 0.65; 95% CI 0.52 to 0.81; 17 RCTs; 5,112 infants). Comparison between use of probiotics and placebo or no treatment showed that there was no statistical difference in the incidence of nosocomial sepsis (RR 0.91; 95% CI 0.80 to 1.03; 19 RCTs; 5,338 infants). The authors concluded that use of enteral probiotics seemed to be associated with reduction of severe NEC and mortality among premature infants. For further details, and to check the form of probiotics used in each study, refer to the original abstract, available at: http://onlinelibrary.wiley.com/doi/10.1002/14651858.CD005496.pub4/full.

#### Postoperative recurrence of Crohn’s disease

The objective of the review^21^ was to evaluate the medical therapies for prevention of postoperative recurrence of Crohn’s disease. It included 23 RCTs, but only 4 RCTs were about probiotics. These RCTs found that there was no difference between probiotics and placebo regarding the risk of clinical recurrence (RR 1.41; 95% CI 0.59 to 3.36; 3 RCTs; 213 adults), risk of severe endoscopic recurrence (RR 0.96; 95% CI 0.58 to 1.59; four RCTs; 333 adults) or risk of any endoscopic recurrence (RR 0.98; 95% CI 0.74 to 1.29; 3 RCTs; 213 adults). The authors concluded that there was no difference in effect between use of probiotics and placebo. For information on the other medical therapies included in this review, refer to this link: http://onlinelibrary.wiley.com/doi/10.1002/14651858.CD006873.pub2/full.

#### Preterm labor

The review^22^ assessed the effectiveness and safety of probiotics for prevention of premature labor and delivery and included three RCTs (344 pregnant women) that compared use of probiotics with acid acetic and placebo. The following results were found: use of probiotics reduced the risk of vaginal infection in comparison with the controls (acetic acid or placebo) (RR 0.19; 95% CI 0.08 to 0.48; 88 pregnant women); comparison of use of probiotics plus dietary counselling versus control (placebo plus dietary counselling or placebo only) showed that there was no difference between the groups regarding birth at gestational ages of less than 32 weeks (RR 0.65; 95% CI 0.03 to 15.88; 1 RCT; 238 pregnant women) or birth between the gestational ages of 32 and 37 weeks (RR 3.95; 95% CI 0.36 to 42.91; 1 RCT; 238 pregnant women).

The authors concluded that use of probiotics seemed to be associated with lower risk of vaginal infection during pregnancy and that there was insufficient evidence to support their use for prevention of premature birth. For further details, and to check the form of probiotics used in each study, refer to the original abstract, available at: http://onlinelibrary.wiley.com/doi/10.1002/14651858.CD005941.pub2/full.

## DISCUSSION

This overview found that despite increasing marketing of probiotics, there are still few systematic reviews on the preventive use of probiotics and there is a scarcity of high-quality randomized trials. None of the reviews included in the present study provided high-quality evidence for any outcome.

Many clinical trials assessed in this study showed very low or low quality of evidence. Another point that needs to be noted is the huge variety of probiotics that have been considered in RCTs. This made it difficult to identify the individual effect of each probiotic agent, and also precluded meta-analyses.

Most of the studies focused on gastrointestinal diseases. We found that there were some benefits from use of probiotics, with moderate quality of evidence, regarding their use for decreasing the incidence of antibiotic-associated diarrhea among children^13^ and the incidence of *Clostridium difficile*-associated diarrhea among adults and children.^17^ Other benefits that were observed with very low or low quality of evidence were that use of probiotics decreased the incidence of infections and the number of infectious episodes in patients undergoing liver transplantation.^16^ The benefit of decreased incidence of severe necrotizing enterocolitis and mortality among preterm infants was noted in another review, but the quality of its evidence could not be assessed.^14^

In relation to respiratory diseases, probiotics showed some benefits regarding decreased incidence of upper respiratory tract infections and duration of episodes, the need for antibiotics and missing school due to colds,^15^ and regarding the incidence of ventilator-associated pneumonia in patients receiving mechanical ventilation.^11^ These studies were classified as presenting very low or low quality of evidence, using the GRADE approach.

In three systematic reviews about gynecological and obstetric diseases, we found that there were some benefits in relation to decreasing the rate of gestational diabetes mellitus, decreasing both the birthweight^8^ and the risk of vaginal infection,^22^ although the quality of evidence could not be assessed. One RCT found that there was a benefit in relation to reducing the incidence of eczema among infants with a family history of allergy or food hypersensitivity and among healthy infants.^12^

Despite the potential benefits of probiotics, we did not find any high-quality evidence that could change clinical practice or recommendations for their use. Furthermore, some probiotics may be harmful in groups of patients such as those presenting immunosuppression, severe debilitation and other such conditions. On the other hand, it is important to examine the number needed to treat (NNT) and to analyze the cost-effectiveness of use of probiotics. Goldenberg et al. concluded that the NNT to prevent one case of diarrhea was ten. Thus, in this example, probiotics reduced the number of cases of diarrhea even with only a few patients treated.^13^ To prevent *Clostridium difficile*-associated diarrhea, 29 patients would need to be treated.^17^

Our systematic review has the advantage of the number of studies included, given that the topic of probiotics is currently a matter of debate and that there are uncertainties regarding their effectiveness. Another advantage is that it summarizes the evidence relating to probiotics and their use that has been gathered in the Cochrane Library, which is recognized as the largest database of systematic reviews, given that the information about probiotics is distributed among many studies.

This overview has some limitations. Our search was conducted in a single database, even though the Cochrane Library is recognized as the most important database of systematic reviews. The limited data available is a consequence of the small number of papers, and the low quality of evidence is related to the small sample sizes and bias of the RCTs. Another point that should be noted is the huge variety of prebiotics that have been considered in RCTs, which led to difficulty in identifying the individual effect of each probiotic agent, and also precluded meta-analyses. The NNT was not determined in some reviews, which made it more challenging to analyze cost-effectiveness.

Regarding the implications for practice, our study summarizes the use of probiotics as a preventive intervention for some clinical settings and shows the situations in which there is a real benefit. From this, healthcare professionals can decide when to indicate probiotics for patients and can improve outcomes in their hospitals. For example, probiotics can be used to reduce the incidence of vaginal infection during pregnancy and to decrease the incidence of VAP. On the other hand, probiotics should not be recommended when there is uncertainty about their benefits and harm.

Here, we make it clear that much needs to be done in relation to studying probiotics. Firstly, basic research is needed in order to elucidate the pathophysiological links between different diseases and use of probiotics. Secondly, RCTs with high-quality evidence are needed, with larger sample sizes and better control over variables. Thirdly, research on the cost-effectiveness of use of probiotics needs to be stimulated, because their use must be analyzed in terms of their consequences for health and economic repercussions.

## CONCLUSION

This overview included 16 Cochrane systematic reviews about the use of probiotics as preventive measures within clinical practice. There was little scientific evidence to support the use of probiotics. None of the reviews provided high-quality evidence for preventive action achieved through use of probiotics and each review analyzed only a few randomized controlled trials.
